# Knock‐on community impacts of a novel vector: spillover of emerging DWV‐B from *Varroa*‐infested honeybees to wild bumblebees

**DOI:** 10.1111/ele.13323

**Published:** 2019-06-12

**Authors:** Robyn Manley, Ben Temperton, Toby Doyle, Daisy Gates, Sophie Hedges, Michael Boots, Lena Wilfert

**Affiliations:** ^1^ Centre for Ecology and Conservation University of Exeter Penryn Campus Penryn TR11 9FE UK; ^2^ Department of Biosciences University of Exeter, Streatham Campus Exeter EX4 4QD UK; ^3^ Medical, Veterinary and Life Sciences University of Glasgow Glasgow G12 8QQ Scotland; ^4^ Department of Integrative Biology University of California Berkeley CA 94720 USA; ^5^ Institute of Evolutionary Ecology and Conservation Genomics University of Ulm D‐89069 Ulm Germany

**Keywords:** Bumblebee, community, deformed wing virus, honeybee, indirect disease emergence, spillover, *Varroa destructor*, vector, virus

## Abstract

Novel transmission routes can directly impact the evolutionary ecology of infectious diseases, with potentially dramatic effect on host populations and knock‐on effects on the wider host community. The invasion of *Varroa destructor,* an ectoparasitic viral vector in Western honeybees, provides a unique opportunity to examine how a novel vector affects disease epidemiology in a host community. This specialist honeybee mite vectors deformed wing virus (DWV), an important re‐emerging honeybee pathogen that also infects wild bumblebees. Comparing island honeybee and wild bumblebee populations with and without *V. destructor*, we show that *V. destructor* drives DWV prevalence and titre in honeybees and sympatric bumblebees. Viral genotypes are shared across hosts, with the potentially more virulent DWV‐B overtaking DWV‐A in prevalence in a current epidemic. This demonstrates disease emergence across a host community driven by the acquisition of a specialist novel transmission route in one host, with dramatic community level knock‐on effects.

## Introduction

Emerging infectious diseases are an ever‐present threat to human, animal and plant populations. Disease emergence can be driven by ecological, evolutionary and anthropogenic factors (Woolhouse *et al.* 2005) with the acquisition of novel transmission routes by existing pathogens increasingly recognised as an important driver (Jones *et al.* 2008). As exemplified by the dramatic emergence of Bovine Spongiform Encephalitis (BSE) via contaminated food (Wilesmith *et al.* 1988), novel transmission routes can have a profound impact on the ecology, epidemiology and evolution of infectious diseases, increasing disease prevalence and creating new selection pressures on pathogens such as those apparent in human blood–borne diseases through intravenous drug use (Mathers *et al.* 2008) and blood transfusions (Raghwani *et al.* 2012). Vector‐borne transmission can drastically increase transmission rate and disease prevalence, by transmitting higher numbers of infectious particles and by‐passing infection barriers encountered during direct transmission (Ryabov *et al.* 2014). Transmission by a novel vector may also lead to the evolution of increased virulence (i.e. the harm suffered by the host) both because of higher infectious doses and because vector‐mediated transmission can relax selection on the trade‐off between transmission and virulence (Day 2002).

The risks associated with disease emergence due to novel transmission routes and the spread of disease vectors have been documented (Kilpatrick & Randolph 2012; Carpenter *et al.* 2013; Mysterud *et al.* 2017), but we lack empirical studies to understand how novel transmission routes impact on multi‐host pathogen epidemiology. The existence of multi‐host RNA viruses across pollinator species and the recent, rapid spread of a specialist viral vector, the honeybee mite *Varroa destructor,* provide a unique system to empirically study the impact of a novel vector on multi‐host pathogens.* V. destructor* jumped hosts from the Asian honeybee (*Apis cerana*) to the Western honeybee (*Apis mellifera*) in the early 1900s (Wilfert *et al.* 2016). It provided a new route of virus transmission in *A. mellifera*, directly transmitting viruses into the haemocoel (Ramsey *et al.* 2019), leading to dramatic increases in prevalence and viral load, particularly for deformed wing virus (DWV), a single positive‐stranded RNA virus (Martin *et al.* 2012; Mondet *et al.* 2014). DWV is a viral complex consisting of three characterised variants, DWV‐A, DWV‐B (also known as VDV‐1) and DWV‐C (Mordecai *et al.* 2015), with only DWV‐A and DWV‐B previously found in UK and French bee populations (McMahon *et al.* 2015; Wilfert *et al.* 2016). Wilfert *et al. *(2016) argued that the anthropogenic movement of *A. mellifera* is the source of a globally emerging DWV epidemic, likely driven by the concurrent spread of the *V. destructor* mite. Increased DWV loads in *V. destructor*‐infected hives are associated with colony losses due to increased overwinter mortality (Highfield *et al.* 2009; Berthoud *et al.* 2010; Genersch *et al.* 2010; Dainat *et al.* 2012). This threat to beekeeping is of grave public concern: honeybees not only produce honey and are important for our cultural heritage (Potts *et al.* 2016) but also pollinate wildflowers and crops.

In addition to the direct effects of the new transmission route on disease in honeybees, there is the potential for indirect disease emergence into the wider community. While *V. destructor* exclusively parasitises honeybees*,* DWV is a multi‐host virus prevalent across wild bee populations (Evison *et al.* 2012; Levitt *et al.* 2013; Fürst *et al.* 2014; McMahon *et al.* 2015), pathogenic to *B. terrestris* as well as honeybees (Fürst *et al.* 2014). The shared use of flowers is a known route of disease transmission within and between pollinator species (Durrer & Schmid‐Hempel 1994; Graystock *et al.* 2015; Manley *et al.* 2015). While *V. destructor* (hereafter referred to as ‘*Varroa*' for simplicity) has invaded the entire European mainland, several *Varroa*‐free island refuges remain off the British Isles and French coast. This creates a natural experiment to study how the acquisition of a novel specialist transmission route affects multi‐host pathogen epidemiology and ecology. We compared the prevalence, viral load and genotype of DWV in honeybees and bumblebees on these *Varroa*‐free refuges, with matched *Varroa*‐present mainland and island sites to show that a change to host–parasite ecology in a single host, in this case the invasion of a novel viral vector in honeybees, has community‐level effects on transmission dynamics in a multi‐host pathogen system. Through extensive sequencing and phylogenetic analysis of viral genotypes across pollinator hosts and sites, we find support for DWV spillover from honeybees to bumblebees and identify DWV‐B as the current dominant viral genotype in the UK and France that has experienced a recent bottleneck and subsequent exponential expansion, which may be driven by *Varroa*‐mediated transmission.

## Materials and methods

We collected 355 *A. mellifera*, 281 *B. pascuorum*, 640 *B. terrestris* and 38 *B. lucorum* (differentiating between *B. terrestris/lucorum* via an mtDNA length polymorphism; Table S1) from 12 sites across England and France, between June and August 2015 (Table S2). The sites comprised four *Varroa*‐free islands (Ushant, L'Hostis 2017; Alderney, Isle of Man and St Mary's of the Isles of Scilly, FERA 2005); three *Varroa*‐present islands and five *Varroa*‐present mainland sites (FERA 2005) (Figure S1; Table S2)*.* Bees were collected from a 1 × 1 km area while foraging on flowers, kept alive individually on wet ice, before transfer to −190° C on the day of collection (or within 48 h for Belle‐Ile and Jersey, as it was not possible to transport a dry shipper to these islands). All samples were subsequently stored at −80° C.

We macerated each bee gut individually in 200 μL of insect ringer solution to allow screening for gut parasites. DNA was extracted from 35 μL of gut solution using Chelex® 100 resin (Biorad). For RNA extractions, we used 80 μL of this gut solution as well as half the head and thorax of individuals (bisected laterally), using 1.3 mL Trizol© (Invitrogen, Carlsbad, CA, USA) for homogenisation in a tissue lyser following the manufacturer's instruction, using bromo‐chloropropane for phase separation. The RNA was resuspended in 400 µL nuclease‐free water. We converted 2 µL of RNA into first‐strand cDNA using GoScript™ Reverse Transcriptase and random hexamer primers, using RNasin® to prevent RNA degradation. To determine the prevalence of DWV‐A, DWV‐B and *N. bombi* and *N. ceranae*, cDNA and DNA was diluted 1:10 prior to PCR. We identified positive samples by PCR using GoTaq® DNA Polymerase (Table S1). To confirm the multiplex *Nosema* PCR bands were species‐specific, we sequenced samples of each (Table S1). We collected microsatellite data of all bumblebee samples to assess if relatedness affected disease prevalence (methods S1).

All DWV‐A‐positive samples (*N* = 94) and 10 randomly chosen DWV‐B‐positive samples per site/species (or total number where sample size was below 10) (*N* = 184) were analysed by qRT‐PCR. We measured nucleic acid quality (all samples had a 260/280 nm ratio between 1.8 and 2.1 (Nanodrop™ 2000 spectrophotometer)) and concentration (Qubit™ Fluorometer) for each individual. We synthesised cDNA from 400 ng of RNA template using GoScript™ Reverse Transcriptase and diluted it 1:10. Duplicate reactions were performed for each sample on a Stratagene machine (Mx3005P) using GoTaq® qPCR Master mix for dye‐based detection (Promega, Table S3). Two no‐template negative samples containing RNase‐free water were run per plate. Absolute quantification was calculated using duplicate eight‐point standard curves of plasmid DNA in a 1 : 10 serial dilution on each plate (Method S2). DWV is a positive strand virus whose negative strand is only present during virus replication; thus, the detection of the negative strand is a strong indicator of a true infection (de Miranda & Genersch 2010). We conducted reverse transcription with tagged virus‐specific forward primers to target the negative strand exclusively (Table S3). We adapted the qPCR assays above using tagged primers to detect replicating virus across our samples: we tested all positive *Bombus* samples (DWV‐A *n* = 18; DWV‐B *n* = 49) and a randomly chosen subset of 10 virus‐positive *Apis* samples for each virus, for the presence of the negative strand (Methods S3, Table S3).

Following Wilfert *et al. *(2016), all individuals identified as positive for DWV‐A or DWV‐B by the prevalence PCR were assayed by PCR for four genomic fragments; L‐protein (*lp*), *vp3*, *helicase* and RNA‐dependent RNA polymerase (*RdRp*) (Table S4), purified using Exonuclease 1 and Antarctic phosphatase (NEB) and sequenced using Big Dye Terminator v3.1 (Applied Biosystems). We manually inspected sequences in Geneious® (v.6.8); only high‐quality (< three ambiguous base pairs), non‐heterozygous sequences of a fragment‐specific minimum length were included in further sequence analysis. Not all fragments from all samples were amplified successfully (of 294 DWV‐B positives, N*_lp_* = 116 (329 bp), N*_vp3_* = 195 (240 bp), N*_helicase_* = 144 (239 bp), N*_rdrp_* = 142 (294 bp)); thus, distinct data sets were used for each genomic fragment, optimising information by maximising the alignment length while keeping as many samples as possible (Table S5). Note that there were too few DWV‐A sequences to pursue population genetic and phylogenetic analyses for this viral genotype. The sequences are deposited in Genbank (MG264907‐MG265503). We created alignments using Geneious® (v.6.8) by mapping the sequences to DWV‐A and DWV‐B reference sequences (NC_004830 and NC‐006494), assigning each sequence to a viral genotype. To maximise the genetic information, we concatenated the DWV‐B sequences from all samples that amplified across all four fragments (*n* = 58, 1108 bp). For recombination analyses, see Method S4a.

We carried out phylogenetic analysis for individual and concatenated DWV‐B fragment alignments (Table S5) in Beast 1.8 following methods described in Wilfert *et al. *(2016); full details in methods S5 and Table S6. We produced Maximum Clade Credibility (MCC) trees (TreeAnnotator (v1.8.4)) to infer host ancestral state probabilities. Phylogenetic trees were produced for each alignment using MrBayes 3.2.6. We produced a median joining phylogenetic network for the concatenated fragment using PopArt (v.1.7). Using DNASPv5.10.1 (Librardo & Rozas 2009), we calculated Tajima's D, Kst (Hudson *et al.* 1992) and the nearest neighbour statistic S_NN_ (Hudson 2000).

We pooled 1000 ng of RNA from 30 *A. mellifera* and 60 *B. terrestris,* from two *Varroa‐*free island sites (Ushant and the Isle of Man) and their paired *Varroa‐*present mainland sites (Le Conquet and Liverpool), to create eight populations for SMRT sequencing. As *B. terrestris* was rare on Ushant, only 13 individuals were in this pool. Full‐length cDNA libraries were prepared using Clontech SMARTer PCR cDNA Synthesis Kit and the BluePippin System. The PacBio Template Prep Kit was used to generate SMRTbell™ libraries, which were sequenced on the PacBio System by Exeter Sequencing Service. Non‐chimeric reads from each pool were mapped against their respective host species genomes using BWA (Li & Durbin 2009) (v. 0.7.12) with the following parameters: ‘bwa mem ‐x pacbio' to remove host‐derived sequences. Remaining reads were mapped against all sequenced bee RNA viruses and 23 novel bumblebee viruses (Pascall *et al.,* 2018) (Table S7). Reads mapping to the genomes of DWV‐A, B and C were extracted for recombination analysis (methods S4b).

All statistical analyses were carried out in RStudio (v0.99.896) (R Core Team 2018). *B. lucorum* samples were excluded from prevalence analyses because of low sample size (*n* = 38), as was the single *B. pascuorum* from Quiberon. True prevalence with 95% confidence intervals was calculated to account for assay efficiency and sensitivity, conservatively set at 95% (Reiczigel *et al.* 2010) using the R library epiR v.0.9‐82 (Stevenson 2018) and the function epi.prev; confidence intervals are calculated based on Blaker (2000). To examine if disease prevalence was affected by *Varroa* presence, we used generalised linear mixed models (GLMMs) with DWV‐B and *Nosema* spp (*N. bombi* and *N. ceranae*) prevalence tested in separate models, with binomial error distribution and logit link function, using the lme4 package (v1.1–12) (Bates & Sarkar 2006). Full models included three‐way interactions between the fixed effects *Varroa* presence, species (a factor with three levels: *A. mellifera, B. terrestris* and *B. pascuorum*) and island/mainland location, with latitude and sunshine hour duration as additional fixed effects; field site and individual were included as random effects (individual was added to account for overdispersion in the model (Harrison 2014)). Latitude and sunshine hours provided a proxy for favourable disease transmission conditions (Fürst *et al.* 2014); sunshine hours were calculated as the mean sunshine hours from monthly data between March and July 2015 collected from MET office data (*pers comms*) and Meteo France (http://www.meteofrance.com/climat/france). The minimum adequate model was identified through the comparison of models using anova and removal of non‐significant terms. To investigate if viral load was affected by *Varroa* presence, we ran GLMMs with Gamma error distribution and reciprocal link function. Viral load data varied across orders of magnitude from 10^3^ to 10^10^; thus, these data were log transformed. *Varroa*‐free refugia only exist on islands; thus, it was necessary to also sample on *Varroa*‐present islands, as well as paired *Varroa*‐present mainland sites, to test a possible island effect on disease prevalence. To test for the effect of an island location, we ran models on reduced data sets (1) comparing island sites with and without *Varroa* and (2) comparing *Varroa*‐present islands and mainland sites.

## Results

*Bombus* and *Apis* individuals were predominantly infected with DWV‐B (true prevalence: DWV‐A = 1.93% (95% CI 0.5–3.6%) and DWV B = 19.70% (CI 17.2–22.3), test of proportions χ^2^ = 128.54_1_, *P* < 0.001). We detected virus replication of DWV‐A in 11.1% (*n* = 18) and DWV‐B in 2.04% (*n* = 49) of all virus‐positive *Bombus* samples; and replication of DWV‐A in 30% (*n* = 10) and DWV‐B in 10% (*n* = 10) of a subsample of virus‐positive *Apis* samples. The negative‐strand assays are highly conservative and can only confirm replication occurs in principle, rather than quantify or exclude it. DWV‐A was notably absent from samples collected from *Varroa‐*free sites apart from one *B. terrestris* individual on the Isle of Man (Figure S2). PacBio single molecule RNAseq data confirmed the prevalence of DWV‐B in PCR results was not an artefact of potential primer bias. Of 20,578 non‐chimeric PacBio reads greater than 1000 bp, 20,560 (99.9%) mapped to DWV‐B, with only 18 mapping to DWV‐A and none to DWV‐C. We therefore focus analysis on the more prevalent DWV‐B.

**Figure 1:**
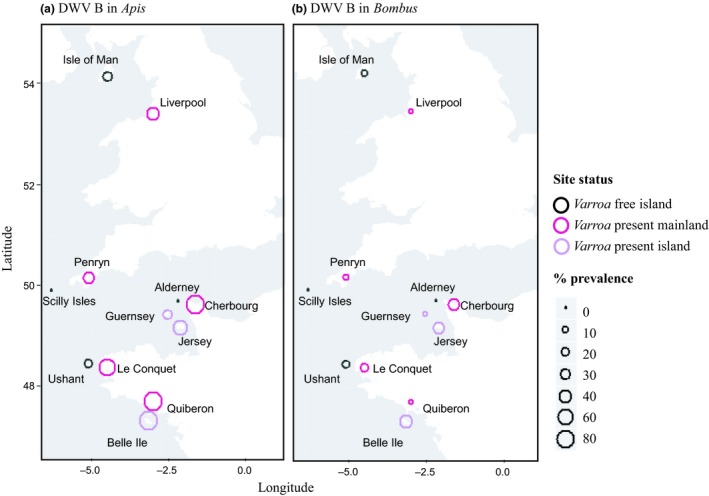
The prevalence of DWV‐B mapped by pollinator genus (a) *A. mellifera, *(b)* Bombus* spp.), location and *Varroa* presence/absence. *Varroa*‐free sites are black, *Varroa*‐present islands are light purple and *Varroa*‐present mainland sites are fuchsia pink. Prevalence is indicated by the size of the circles as shown in the key.

**Figure 2:**
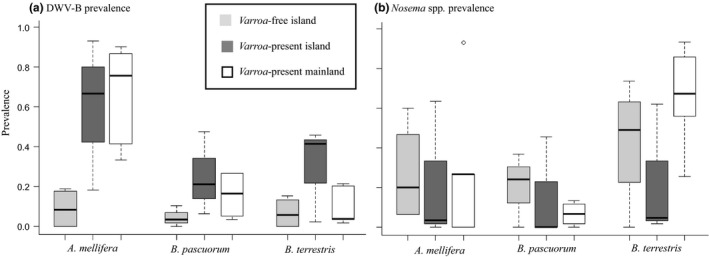
The prevalence (with 95% confidence intervals) of DWV‐B (a) *Nosema* spp. (b) by host species and *Varroa*‐free (light grey), *Varroa*‐present island (dark grey) and *Varroa*‐present mainland sites (white).

Prevalence screens show DWV‐B to be predominantly a honeybee virus whose prevalence is increased in both honeybees and bumblebees by the presence of *Varroa* (GLMM: estimate ± SE = 3.04 ± 1.14, *P* = 0.008, Table S8). Across our 12 populations (Fig. 1), DWV‐B was highly prevalent in honeybees with an average of 45.1% (*n* = 355) positive for the virus, with lower prevalence of 9.5% in *B. pascuorum* (*n* = 280) and 10.0% in *B. terrestris* (*n* = 641) (Fig. 2). *Varroa* presence is a significant predictor of the prevalence of DWV‐B for all species, predicting a *c*. 7‐fold increase in *A. mellifera* and *c*. 19‐fold increase in both *B. terrestris* and *B. pascuorum* (Table S10). There was no evidence that honeybees and bumblebee species respond differently to *Varroa* presence, as the interaction between species and *Varroa* did not contribute to the model fit (model comparison anova: χ^2^ = 3.96_2_, *P* = 0.14), and was thus removed from the model. Importantly, given that all *Varroa‐*free sites in this study are islands, there was no evidence that island/mainland location influenced DWV‐B prevalence (GLMM: estimate ± SE = −0.59 ± 1.03, *P* = 0.57, Table S8), even though there is a significant interaction between island and host species (Table S8). Furthermore, comparing between *Varroa‐*present islands and mainland sites, island/mainland location has no effect on DWV‐B prevalence (GLMM: estimate ± SE = −0.15 ± 0.92, *P* = 0.87). When comparing island sites with and without *Varroa,* the significant effect of *Varroa* on DWV‐B prevalence is confirmed (GLMM: estimate ± SE = 3.31 ± 1.51, *P* < 0.028). Latitude and sunshine hours were not significant and excluded from all final models (Table S8). Wild bumblebee colony density was similar across all sites, with an average of 1.18 and 1.15 individuals per colony per site for *B. terrestris* and *B. pascuorum* respectively (χ^2^ = 2.16_2_, *P* = 0.34; Kruskal–Wallis rank sum test) (Table S9).

To further confirm that the presence of *Varroa* itself, rather than a higher degree of isolation of *Varroa‐*free refugia, is driving the increase in DWV, we investigated the prevalence of microsporidian *Nosema* parasites (*N. bombi* and *N. ceranae*)*. Varroa* presence did not influence *Nosema* prevalence (GLMM: estimate ± SE −1.16 ± 0.82, *P* = 0.16). There was no evidence that island/mainland location influenced *Nosema* prevalence (GLMM: estimate ± SE = −1.37 ± 0.80, *P* = 0.09). The prevalence of these parasites was affected by host species (Fig. 2) and duration of sunshine hours (Table S11), with the emerging *N. ceranae* only found in honeybees (Fig. S3).

**Figure 3:**
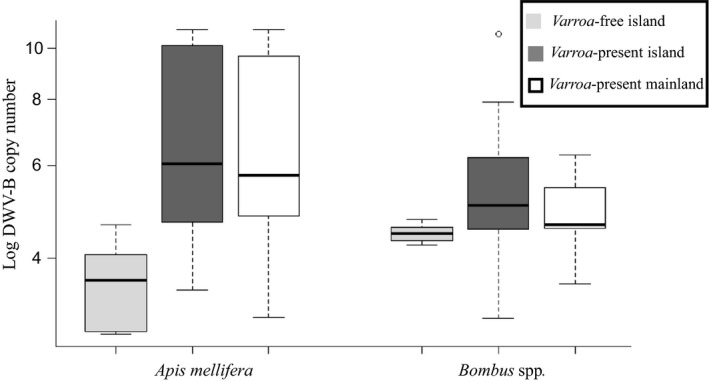
DWV B viral load (with 95% confidence intervals) by host species and *Varroa*‐free (light grey), *Varroa*‐present island (dark grey) and *Varroa*‐present mainland sites (white).

Quantitative analysis of viral titres confirmed that while honeybees overall show higher titres, the presence of *Varroa* not only significantly increases the mean titre of DWV‐B in honeybees but also in bumblebees (Fig. 3; Table S8): For *A. mellifera* the predicted mean DWV‐B viral load is 1.5 × 10^4 ^viral particles per bee on *Varroa‐*free sites, compared to 4.2 × 10^6 ^in *Varroa‐*present sites (*P* < 0.001). In both bumblebee species, the titre also increased by more than one order of magnitude in the presence of *Varroa* (from 3.7 × 10^3^ to 1.7 × 10^5^, and 2.4 × 10^3 ^to 7.1 × 10^4 ^viral particles per bee, for *B. terrestris* and *B. pascuorum* respectively).

**Figure 4:**
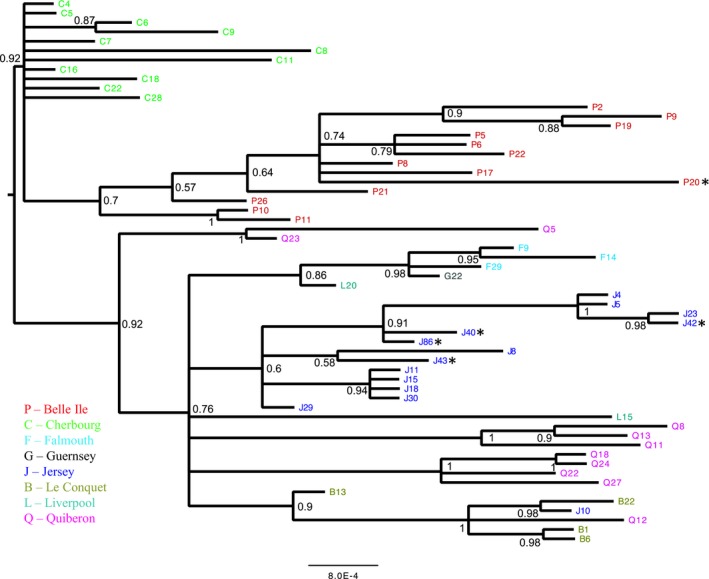
Bayesian phylogeny of DWV‐B. DWV‐B sequences isolated from *A. mellifera* and B. *terrestris*. Sequences are comprised of four concatenated fragments of DWV‐B (*n* = 58, 1108 bp)). The tip labels are coloured by geographic location (see key); host species are *A. mellifera* unless indicated by an asterisk (*B. terrestris*).

Testing the genetic diversity and structure of DWV‐B among species, we find that the same genotypes circulate in honeybees and bumblebees, with no evidence for population differentiation within the concatenated genomic fragments based on the proportion of genetic variation between species (Kst_concat_ = 0.005, *P* > 0.05). There is, however, evidence for geographic population structure (Kst_concat_ = 0.245, *P* < 0.001); samples that are genetic nearest neighbours often come from the same population (Snn_concat_ = 0.657, *P* < 0.001), as can be seen in a Bayesian phylogenetic tree (Fig. 4). The results for each individual fragment corroborate the results of the concatenated fragments data (Table S12 and Figure S4). Successful sequencing of DWV‐B‐positive samples from *Varroa‐*free sites was minimal because of low titre (1 for the *lp* fragment and 4 for the *Vp3* fragment) and was thus excluded from the concatenated data set; however, analysing this small data set, we found no evidence for population differentiation between *Varroa‐*free and *Varroa‐*positive sites (Kst_Vp3_ = 0.0001, *P* > 0.05), as supported by the *lp* and *Vp3* MrBayes trees (Figure S4). Phylodynamic reconstruction in Beast 1.8 (Drummond *et al.* 2012) identified *A. mellifera* as the ancestral host of DWV‐B in these populations for the concatenated fragments (state probabilities: *A. mellifera* = 99.9%, *B. terrestris* = 6.666 × 10^−4^; Figure S5), reflecting the true prevalence of this virus in the sampled populations.

A sliding 50 bp window analysis identified few putative recombination events within the PacBio reads that mapped to a DWV genome: Of a total of 20,578 reads, 61 contained windows that had a best hit to more than one DWV genome. When a cut‐off minimum of three consecutive windows (150 bp) was applied, only five reads (all isolated from *A. mellifera* at the *Varroa*‐present Liverpool site) were identified as putative recombinants. Only three of these showed a consistent transition from one DWV genome to another with the point of recombination within the *rdrp* gene (Figure S6). Using RDP4 (Martin *et al.* 2015) (methods S4a), we also found evidence for recombination via Sanger sequencing in the *lp*‐gene region, with 13 of 116 sequences showing evidence of recombination using multiple algorithms in RDP4.

**Figure 5:**
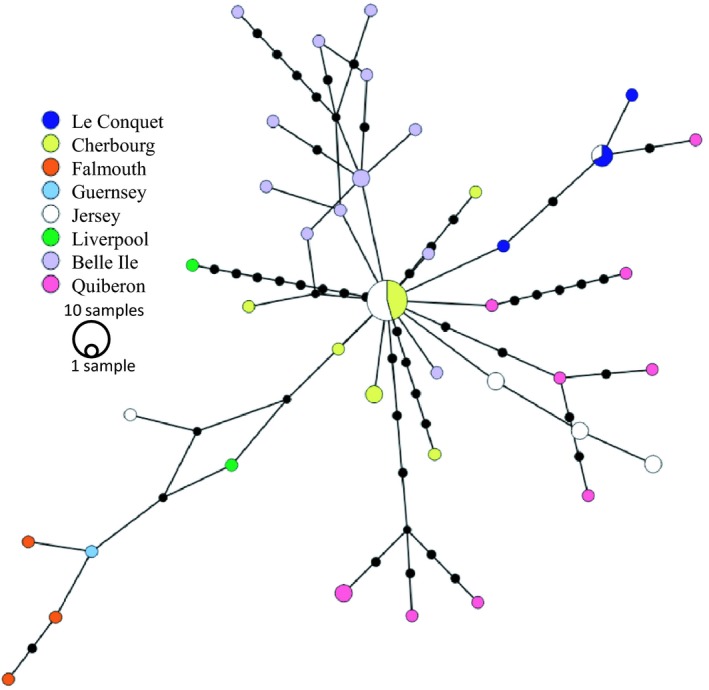
Median joining phylogenetic network of concatenated DWV‐B sequences (*n* = 58), showing a star‐shaped network as expected following a rapid expansion. The colours represent sampling location, the size of the node represents the number of samples with the same sequence and the black dots on branches show the number of mutations between nodes.

The low genetic diversity of the DWV‐B phylogeny (π = 0.0051, with 71 polymorphic sites of 1108 sites of the concatenated fragments, examined over 58 sequences, (Table S5) and a star‐shaped network of sequence similarity (Fig. 5), suggests a recent bottleneck and subsequent exponential expansion. This result is supported by phylogenies for the concatenated fragments (Fig. 4 and S5). The low levels of population structure enabled us to combine sequences from across populations to investigate the past demography of the virus. We found a large excess of rare variants compared with the neutral model, suggestive of an expanding population after a bottleneck (Tajima's D for DWV‐B concatenated fragments = −2.276, *P* < 0.001, 95% CI: −1.62 to 1.92). This is supported for each individual fragment across populations (Tajima's D for *lp* = −2.187, *vp3* = −2.475, *helicase* = −2.255, *rdrp* = −2.498; *P* < 0.001). Phylodynamic reconstruction also supports this recent expansion: the most recent common ancestor for the concatenated fragments dates back only 6 years (mean root height for concatenated fragments = 5.7 (95% HPD 2.0–10.3), which corroborates findings for individual fragments (Table S13). All DWV‐B individual and concatenated fragments showed exponential growth, with doubling rates estimated to be less than a year (doubling rate for concatenated fragment = 0.73 years (95% HPD 0.39–2.39). We find evidence of a strong expansion of DWV‐B, with honeybees in *Varroa‐*present locations showing an average DWV‐B prevalence of 64.8%, compared to DWV‐A at 29.2% (*n* = 233).

## Discussion

By taking advantage of the natural experiment created by the spread of the novel viral vector *Varroa* in honeybees, we have shown clear knock‐on community epidemiological impacts that result from ‘novel transmission route' disease emergence in a single host. The comparison of *Varroa*‐free and matched *Varroa*‐present sites demonstrates clearly that *Varroa* increases the prevalence and titre of DWV in its host, the honeybee, and that this causes spillover into wild bumblebees. There is higher prevalence and viraemia in bumblebees in the presence of *Varroa,* even though the mite only infests honeybees. Genetic analysis confirms that the same genotypes of virus are circulating in all the bees, which is consistent with DWV spilling over from the honeybees. In direct comparison, the prevalence of *Nosema* species *(N. ceranae* and *N. bombi*), an emerging bee disease (Fürst *et al.* 2014) whose oral–faecal transmission is not linked to *Varroa*, is unaffected by the presence of this honeybee ectoparasite. These results support reports from Hawaii of increased DWV prevalence in two sympatric pollinators (*Poliste*s wasp species and solitary bee *Ceratina smaragdula*) (Santamaria *et al.* 2018) and a honeybee predator, *Vespula pensylvanica* (Loope *et al.* 2019) following the invasion of *V. destructor* (Martin *et al.* 2012)*.*

Data are scarce on DWV prevalence, titre and diversity preceding the arrival of *Varroa* into western honeybee populations, especially in relation to wild bees. However, low‐level and benign DWV infections likely circulated in *A. mellifera* (de Miranda & Genersch 2010; Genersch & Aubert 2010) and are documented from Hawaii (Martin *et al.* 2012) and New Zealand (Mondet *et al.* 2014). Phylogenetic analyses suggest that the current DWV epidemic is a re‐emergence of the virus driven by anthropogenic movement of honeybees, coinciding with the invasion of a novel vector, the *Varroa* mite (Wilfert *et al.* 2016). The primary mechanism behind the dramatic effect of *Varroa* on DWV re‐emergence is likely the increase in titre and transmission events through the direct injection of viruses into the honeybee haemocoel while feeding on the fat body (Ramsey *et al.* 2019). It is also possible that the physical feeding activity of the mite itself (Kuster *et al.* 2014) or immunosuppression of the bee (Nazzi *et al.* 2012) could cause the increase in DWV. In addition, it has been suggested that *Varroa* drives selection on DWV leading to loss of variant diversity, and resulting in the dominance of a single master variant (Martin *et al.* 2012). In contrast to Martin *et al. *(2012), we did not find greater viral genotype diversity without *Varroa* (based on our PacBio viral sequence mapping results, which confirm the predominance of DWV‐B).

A surprising finding of our study is that DWV‐B is the dominant variant, rather than the globally distributed DWV‐A variant implicated in the current worldwide DWV epidemic (Martin *et al.* 2012; Wilfert *et al.* 2016). Phylogenetic analyses suggest that DWV‐B emerged in our European populations within the last decade (since 2009) and expanded exponentially after this genetic bottleneck; this result is supported by a significant excess of rare mutations in these populations. A series of surveys across similar locations provides further support for a recent exponential spread of DWV‐B: in 2009, DWV‐A dominated (Wilfert *et al.* 2016); in 2011, DWV‐B prevalence was high but equal to that of DWV‐A (McMahon *et al.* 2016); and in 2015 (present study), we find that DWV‐B is dominant. It is possible that this change in genotype prevalence is caused by *Varroa*‐mediated transmission exerting strong selection on DWV (Martin *et al.* 2012; Gisder *et al.* 2018), favouring DWV‐B. Evidence from laboratory competition assays simulating transmission via *Varroa* show that DWV‐B replicates to higher titres than DWV‐A in honeybee adults (at 9 days post‐infection) (McMahon *et al.* 2016) and in eclosing bees (6–7 days after inoculation as pupae) (Tehel *et al.* 2019). Here, we find both DWV‐A and DWV‐B to be more prevalent with higher intensities where *Varroa* is present, indeed with DWV‐A being almost absent from *Varroa‐*free sites, and thus, our data do not per se support the hypothesis that the acquisition of *Varroa* has caused this change in the prevalence of viral genotypes. We also find little genetic variation across the DWV‐B populations, with no population structure by host species or *Varroa* presence, and only modest population structure by location. However, pathogens can spread ahead of their vector if a host can carry, replicate and transmit viruses, potentially obscuring any role of *Varroa‐*mediated selection in the field. As our populations have endured over 20 years of *Varroa* infestation (FERA 2005), we might therefore find a similar diversity and the prevalence of viral variants on *Varroa*‐free islands through spillover via trade, travel, deliberate and accidental transportation, and possibly migration, of infected competent pollinator hosts across these highly connected locations over time.

*Varroa‐*mediated transmission has also been suggested to select for DWV‐A/B recombinants, with the non‐structural proteins including the replication machinery typically provided by DWV‐A and the capsid genes typically provided by DWV‐B (Ryabov *et al.* 2014), a pattern also found in apiaries in the UK (Moore *et al.* 2011; Wang *et al.* 2013) and Israel (Zioni *et al.* 2011). However, our results provide little support for this pattern via Pacbio or Sanger sequencing. We found no evidence of previously reported recombination within the N‐terminal region of the helicase gene (Dalmon *et al.* 2017). Instead, we found limited evidence of recombination within the *rdrp* and* lp* genes, similar to reports from the UK (Moore *et al.* 2011; Wang *et al.* 2013). While these data cannot systematically address the question of genome‐wide recombination, our results give little evidence for an important role of recombination in the current spread of DWV.

Interspecific transmission clearly occurs because the same genotypes are found across all bee species. We find DWV‐B to be far more prevalent than DWV‐A in bumblebees across our samples, which is particularly concerning in light of McMahon *et al. *(2016) demonstrating in laboratory studies that DWV‐B is a more virulent genotype than DWV‐A in adult *A. mellifera*, with unknown effects on *Bombus* species. Field studies also show that DWV‐B infection is linked to overwinter hive mortality (Natsopoulou *et al.* 2017). Furthermore, DWV‐B has recently been shown to have dramatically increased in prevalence in the USA, from only 2.7% of colonies screened in 2010 (*n* = 75) to 66% of apiaries screened (*n* = 603) in 2016 (Ryabov *et al.* 2017).

We present compelling evidence, in line with other studies (Fürst *et al.* 2014; Wilfert *et al.* 2016), that *A. mellifera* is both the ancestral and reservoir host for DWV. Significantly higher prevalence of both DWV variants in *A. mellifera* compared to bumblebees found in this study is consistent with this hypothesis. The prevalence of DWV in *A. mellifera* has been linked to prevalence in bumblebees, strongly suggesting spillover between managed honeybees and wild pollinator populations (Fürst *et al.* 2014). Importantly, we show that DWV‐A and B cause true infection in both honeybees and bumblebees by the detection of the virus negative strand, which is only present during replication for a positive‐sense RNA virus (Fürst *et al.* 2014; Radzevičiùte *et al.* 2017). Furthermore, the high viral loads across bumblebees, specifically in DWV‐B, combined with the effect of *Varroa* presence increasing DWV viral load in bumblebees, suggest that we are detecting true DWV infections in bumblebee hosts. However, we cannot confirm if higher viral loads in bumblebee hosts are simply the result of spillover of higher viral loads also recorded in sympatric honeybees, or if the recently emerged DWV‐B variant that dominates in our samples is better able to replicate to high levels in bumblebee hosts. As the same genotypes are found in *Varroa‐*free sites at lower prevalence and titre, spillover is the likely explanation.

Infectious diseases, and their interactions with other anthropogenic drivers of species declines, are a concern for the sustainability of wild and managed pollinator populations (Goulson *et al.* 2015). The levels of *Varroa* infestations correlate with DWV viral titres in honeybees (Nazzi *et al.* 2012) and therefore their potential to transmit the virus to wild pollinators. Thorough *Varroa* and pathogen control by beekeepers is essential for the protection of wild pollinators from disease. It further highlights the importance of establishing vector‐free *refugia* both for this pollinator parasite, and for multi‐host pathogen systems in general, for maintaining future biodiversity.

The spread of disease vectors through global change poses the risk of disease emergence. The introduction of avian malaria and its mosquito vector to Hawaii, for example, has led to a dramatic reduction in abundance and diversity of Hawaiian land birds (van Riper *et al.* 1986). Vector range expansions, facilitated by climate change, pose a significant risk to humans, wildlife and plants alike, as illustrated by the increasing spread of arboviruses such as Bluetongue in Europe in line with the range expansion of their vectors (Purse *et al.* 2005). This work has so far focussed on systems where all potential hosts acquire the vector. Here, however, we show that a specialist vector on one host species can change disease epidemiology throughout a multi‐host pathogen community. While the emergence of novel vectors may be a rare event, global change and direct human interference may frequently lead to the establishment of novel transmission routes within and between species, from the use of blood transfusions to alterations in animal food chains. Our work demonstrates that seemingly isolated changes within one host may impact epidemiology at a community level with important knock‐on effects due to spillover.

## Author contributions

The study was designed by LW and RM, with input from MB. RM led the fieldwork, with contribution from DG. RM, DG, SH and TD performed lab work. BT contributed bioinformatics analysis of SMRT data. RM and LW analysed the data. The paper was written jointly by RM, MB and LW, with input from all authors.

## Data Availability

The Sanger sequences that support the findings of this study have been deposited in GenBank with virus accession codes MG264907‐MG265503 and *Nosema* accession codes MK942707‐MK942712; SMRT reads have been archived in NCBI's Sequence Read Archive with BioProject accession number PRJNA542789. Prevalence and qPCR data that support the findings will be available from the Dryad Digital Repository: https://doi.org/10.5061/dryad.70jt240.
