# Supplementary material for: Knock‐on community impacts of a novel vector: spillover of emerging DWV‐B from Varroa‐infested honeybees to wild bumblebees
Source: Ecol Lett. 2019 Jun 12;22(8):1306–15. doi: 10.1111/ele.13323 (PMC6852581; doi:10.1111/ele.13323)
Supplement: Supplementary file 1 [file ELE-22-1306-s001.docx]

## Extended data

### Supplementary tables

Table S1: Primers and protocols used to differentiate the *B. terrestris/lucorum* complex, and detect the prevalence of pathogens DWV-A and DWV-B; and to detect prevalence of *Nosema apis, N. bombi* and *N. ceranae,* and to sequence them

| Target | Primer name | Sequence | Amplification program | Amplicon (bp) | Reference |
| --- | --- | --- | --- | --- | --- |
| *B. terrestris/*  *B. lucorum* complex | BBM1IGSF  BBM1IGSR | GGAGCAATAATTTCAATAAATAG  AARTTCAAAGCACTAATCTGC | 15 s at 95C 15 s at 55C  45 s at 72C  X38 cycles | 180  210 | R.Schmid-Hempel (*pers comm*) |
| DWV-A  DWV-B | DWV F1a  VDV F1a  DWV-VDV 7aR | ggaaacatctggaattagcgacaA  GAAAACATTTGGAATTAGCAACGAC  AATCCGTGAATATAGTGTGAGG | 10 s at 95C,  30 s at 60C,  30 s at 72C  X35 cycles | 360  360 | McMahon *et al.* (2015) |
| *Nosema* Multiplex PCR (for prevalence) | Mnapis_F  MnBombi-F  Mnceranae_F  Mnuniv_R | GCATGTCTTTGACGTACTATG  TTTATTTTATGTRYACMGCAG  CGTTAAAGTGTAGATAAGATGTT  GACTTAGGAGTAGCCGTCTCTC | 30 s at 95C  30 s at 56C  60 s at 72C  X35 cycles | 224  171  143 | Fries *et al.* (2013) |
| *Nosema* all species (for sequencing) | SSUrRNA-f1b  SSUrRNA-r1b | CACCAGGTTGATTCTGCCT  TGTTCGTCCAGTCAGGGTCGTCA | 60 s at 94C  30 s at 64C  30 s at 72 | 545 | Tay *et al.* (2005) |

Table S2. The town and location of field site, and the total number of each bee species collected from each site. Note. *Bombus terrestris* and *B.* *lucorum* are cryptic and required molecular identification post-collection

| Location | *Varroa*-status | *A. mellifera* | *B. terrestris* | *B. lucorum* | | *B. pascuorum* |
| --- | --- | --- | --- | --- | --- | --- |
| Guernsey (St Peter Port) | *Varroa*-present-islands | 22 | 45 | | 15 | 32 |
| Jersey (St Helier) |  | 30 | 59 | | 1 | 33 |
| Belle-Ile (Le Palais) |  | 29 | 59 | | 1 | 19 |
| Alderney (St Anne) | *Varroa*-free-islands | 30 | 57 | | 3 | 30 |
| Isles of Scilly (St Mary’s) |  | 30 | 60 | | 0 | 0 |
| Ile d’Oeussant |  | 30 | 13 | | 2 | 29 |
| Isle of Man (Douglas) |  | 32 | 53 | | 5 | 29 |
| Quiberon, Brittany | *Varroa*-present-mainland | 30 | 59 | | 0 | 1 |
| Cherbourg (marina area) |  | 30 | 61 | | 3 | 30 |
| Penryn (University campus) |  | 30 | 56 | | 5 | 30 |
| Le Conquet, Brittany |  | 33 | 59 | | 2 | 19 |
| Liverpool, city centre |  | 29 | 59 | | 0 | 29 |
|  |  |  |  | |  |  |

Table S3: Primers and protocols used for qPCR amplification and to create plasmids for standard curves.

| Primer | Sequence | Amplification program | Reference |
| --- | --- | --- | --- |
| DWV_F2*  DWV_R2a* # | TGTCTTCATTAAAGCCACCTGGAA  TTTCCTCATTAACTGTGTCGTTGAT | 10 s at 95C,  30 s at 60C,  X40 cycles  Melting curve profile:  55 – 95C (0.5C per second increments) | McMahon *et al.* (2015) |
| DWV_F1a **  DWV_7Ra** | ggaaacatctggaattagcgacaAA  AATCCGTGAATATAGTGTGAGG |  |  |
| VDV_F2  VDV_R2a # | TATCTTCATTAAAACCGCCAGGCT  CTTCCTCATTAACTGAGTTGTTGTC |  |  |
|  |  |  |  |
| Rp49_qF | AAGTTCATTCGTCACCAGAG |  | de Miranda (2008)  Fürst *et al.* (2014) |
| Rp49_qB  Tag #  DWV_F2_TAG  VDV_F2_TAG | CTTCCAGTTCCTTGACATTATG  AGCCTGCGCCACCGTGG  agcctgcgcaccgtggTGTCTTCATTAAAGCCACCTGGAA  agcctgcgcaccgtggTATCTTCATTAAAACCGCCAGGCT |  |  |

Note * primers were used for DWV-A qPCR assays, while ** primers were used to create the DWV-A plasmid; # primers were used in negative strand qPCR detection assays.

Table S4. Primer sequences, protocols and amplicon product size for four genomic fragments across a) DWV–A and b) DWV-B genomes. Note for DWV-A *rdrp* primers, the genome position is given in brackets as it was not included in the name

a)

| Primer name & genome position | Sequence | Amplification program  35 cycles | Amplicon (bp) | Source |
| --- | --- | --- | --- | --- |
| **DWV–A primers** |  |  |  |  |
| Lp _F1153  Lp_ B1806 | ATTAAAAATGGCCTTTAGTTG  CTTTTCTAATTCAACTTCACC | 30 s at 94C  30 s at 55C  30 s at 72C  X35 cycles | 653 | Wilfert *et al.* (2016) |
|  |  |  |  |  |
| VP3_DWV F1 (3722)  VP3_DWV B1 (4076) | CCTGCTAATCAACAAGGACCTGG  CAGAACCAATGTCTAACGCTAACCC | 30 s at 94C  30 s at 55C  30 s at 72C  X35 cycles | 355 | Genersch (2005) |
|  |  |  |  |  |
| Helicase_6285F  Helicase_6693R | GAGCGTACACTATGGTCAGA  GTTCACGACGCTTACTACAC | 30 s at 94C  30 s at 56.6C  30 s at 72C  X35 cycles | 409 | Berenyi *et al.* (2007) |
|  |  |  |  |  |
| RdRp_F15 (9247)  RdRp_B23 (9697) | TCCATCAGGTTCTCCAATAACGGA  CCACCCAAATGCTAACTCTAAGCG | 30 s at 94C  30 s at 49.2C  30 s at 72C  X35 cycles | 450 | Yue and Genersch (2005) |
|  |  |  |  |  |
|  |  |  |  |  |
| b) |  |  |  |  |
| **DWV–B primers** |  |  |  |  |
| Lp_1_1520F | AAGAAAGTGAAACGGGTGGC | 30 s at 94C  30 s at 62.2C  30 s at 72C  X35 cycles | 437bp | This study |
| Lp_1_1998R | ATTAAGCGCGCCAATTCCTT |  |  |  |
| VP3_1_3707 | CAAGGACCCGGCAAAGTAAG | 30 s at 94C  30 s at 63.3C  30 s at 72C  X35 cycles | 383bp |  |
| VP3_1_4089 | CCATCACGGCAGCGATTAAA |  |  |  |
| Heli_1_6428 | TATGCAGCAGGAATGAACGC | 30 s at 94C  30 s at 59.6C  30 s at 72C  X35 cycles | 318bp |  |
| Heli_1_6745 | TGTAGAACGCTCGTGGACAT |  |  |  |
| RdRp_2_9343 | CGTGCTAGTTTGTTACGGTGA | 30 s at 94C  30 s at 61.6C  30 s at 72C  X35 cycles | 435bp |  |
| RdRp_2_9777 | ACATCCATTTCTTCCCATGTGA |  |  |  |

Table S5: Alignment details: number of sequences and alignment length, genetic diversity (π), number of segregating sites (S) and parsimony informative sites (*PiS*), and the number of sequences from each host species (*A. mellifera, B. terrestris* and *B. pascuorum*). Note, recombinant *lp* samples were excluded from alignments.

| Fragment | N_Sequences_ | Length[bp] | π | *S* | *PiS* | N*_A.mellifera_* | N*_B.terrestris_* | N*_B.pascuorum_* |
| --- | --- | --- | --- | --- | --- | --- | --- | --- |
| *lp* | 116 | 331 | 0.0065 | 30 | 16 | 97 | 16 | 3 |
| *vp3* | 195 | 242 | 0.0015 | 20 | 3 | 133 | 47 | 15 |
| *helicase* | 144 | 241 | 0.0019 | 14 | 9 | 108 | 31 | 5 |
| *rdrp* | 142 | 294 | 0.0022 | 26 | 6 | 116 | 23 | 3 |
| concatenated | 58 | 1108 | 0.0051 | 71 | 30 | 53 | 5 | 0 |

Table S6. Substitution models for each DWV-B alignment based on jModelTest (Guindon & Gascuel 2003b; Darriba *et al.* 2012a). Results of path sampling maximum likelihood estimator analysis comparing demographic and molecular clock models run in Beast 1.8. Analyses of each model in Tracer showed that a strict molecular clock could not be excluded for any model, and all fragments had significant exponential growth, thus for these models * we override path sampling selection.

| Fragment and traits | Substitution model | Molecular clock | Population demography | | |
| --- | --- | --- | --- | --- | --- |
|  |  |  | Constant | Exponential | GMRF Skyride |
| - - - 1. *lp* | HKY + G | Strict | -1335.06 | **-1317.47** | -1323.90 |
| (no traits) |  | Exponential | -1332.10 | -1313.63* | -1321.31 |
|  |  | Lognormal | -1332.00 | -1319.72 | -1327.38 |
| - - - 1. *vp3* | K80 + G | Strict | -1245.50 | **-1226.50** | -1235.76 |
| (no traits) |  | Exponential | -1245.57 | -1222.91 | -1229.38 |
|  |  | Lognormal | -1241.78 | -1220.23* | -1237.03 |
| - - - 1. *helicase* | HKY + G | Strict | -749.74 | **-743.06** | -750.49 |
| (no traits) |  | Exponential | -749.67 | -745.22 | -750.50 |
|  |  | Lognormal | -751.36 | -744.81 | -751.41 |
| - - - 1. *rdrp* | K80 + G | Strict | -621.20 | **-610.98** | -617.97 |
| (no traits) |  | Exponential | -621.35 | -610.82 | -618.76 |
|  |  | Lognormal | -621.00 | -610.63* | -618.74 |
| - - - 1. concatenated | K80 + G | Strict | -2573.87 | **-2556.42** | -2560.85 |
| (host and site) |  | Exponential | -2573.13 | -2558.87 | -2562.56 |
|  |  | Lognormal | -2573.58 | -2558.83 | -2560.00 |

Table S7. List of reference virus genomes used in BWA to align SMRT sequence reads. * DWV-C sequences can be found at <http://www.ebi.ac.uk/ena/data/view/CEND01000001>

| Virus | Full virus name | Genbank Reference | |  |
| --- | --- | --- | --- | --- |
| BQCV | Black queen cell virus | NC-0003784 | |  |
| SBPV Harpenden | Slow bee paralysis virus | GU93876 | |  |
| SBPV Roththamsted | Slow bee paralysis virus | EU035616 | |  |
| DWV-A | Deformed wing virus A | NC-004830 | |  |
| DWV-B | Deformed wing virus B | NC-006494 | |  |
| DWV-C | Deformed wing virus C | ENA: CEN01000001* | |  |
| SBV | Sacbrood virus | AF092924 | |  |
| ABPV | Acute bee paralysis virus | NC-002548 | |  |
| KBV | Kashmir bee virus | NC-004807 | |  |
| IAPV | Israeli acute paralysis virus | KY243933 | |  |
| CBPV | Chronic bee paralysis virus | NC-010711 and NC010712 | |  |
| LSv 1 | Lake Sinai virus 1 | HQ871931 | |  |
| LsV 2 | Lake Sinai virus 2 | HQ888865 | |  |
| Alpv | Aphid lethal paralysis virus | KJ817182 |  |  |
| AmFv | Apis mellifera filamentous virus | NC- 027925 |  |  |
| **New bumblebee viruses (Pascall *et al.* 2018)**  Acry1  Bloom1  Bloom2  Bloom3  Bou1  Bou2  Bou3  Corn1  Dia1  Dicist_Full  Dicist_Half  Grange1  I1  I2  Mut1  N1  N2  Sac1  Toti1  Toti2  Toti3  Toti4  Wuchang1 | N/A | N/A |  |  |

Table S8: Best model explaining a) DWV-B prevalence (GLMMs with binomial error structure and logit link function) and b) DWV-B viral load (GLMMs with gamma error structure and inverse link function). The predictor state of the intercept is *A. mellifera,* on a *Varroa*-free site. Population of origin was included as a random effect.

| Response | Predictors | Estimate | SE | z-value | P-value |
| --- | --- | --- | --- | --- | --- |
| a) DWV-B prevalence | Intercept | -2.25 | 1.3 | -1.73 | 0.084 |
|  | *B. pascuorum* | -2.80 | 0.41 | -6.70 | <0.001 |
|  | *B. terrestris* | -3.38 | 0.35 | -9.60 | <0.001 |
|  | Island site  *Varroa* presence | -0.59 | 1.03 | -0.57 | 0.57 |
|  |  | 3.04 | 1.14 | 2.67 | 0.008 |
|  | *B.pascuorum*:island | 1.17 | 0.56 | 2.08 | 0.037 |
|  | *B.terrestris*:island | 2.06 | 0.48 | 4.29 | <0.001 |
| b) DWV-B viral load | Intercept | 0.24 | 0.027 | 8.52 | <0.001 |
|  | *B. pascuorum* | 0.055 | 0.002 | 2.66 | 0.0080 |
|  | *B. terrestris* | 0.04 | 0.01 | 3.94 | <0.001 |
|  | *Varroa* presence | -0.089 | 0.026 | -3.30 | <0.001 |

Table S9. The number of individuals sampled, colony number and average number of individuals collected per colony at each sampling location for bumblebee species *B. terrestris* and *B. pascuorum* from microsatellite data. Note, samples of *B. terrestris* from Ushant were not genotyped due to poor DNA quality, and *B. pascuorum* are absent from the Scilly Isles, and only 1 was collected from Quiberon, thus these data were excluded from the dataset. Further, while all samples were analysed, any that failed to amplify were excluded from this dataset.

|  | *Bombus terrestris* | |  | *Bombus pascuorum* | |  |
| --- | --- | --- | --- | --- | --- | --- |
| Location | Individuals (n) | Colonies  (n) | Average (individuals/colony) | Individuals (n) | Colonies  (n) | Average (individuals/colony) |
| Alderney | 33 | 33 | 1 | 30 | 28 | 1.07 |
| BelleIle | 46 | 35 | 1.31 | 18 | 17 | 1.06 |
| Brest | 54 | 41 | 1.32 | 18 | 17 | 1.06 |
| Cherbourg | 40 | 27 | 1.48 | 24 | 18 | 1.33 |
| Falmouth | 46 | 44 | 1.05 | 27 | 26 | 1.04 |
| Guernsey | 36 | 36 | 1 | 25 | 22 | 1.14 |
| IOM | 51 | 41 | 1.24 | 25 | 19 | 1.32 |
| Jersey | 47 | 44 | 1.07 | 23 | 20 | 1.15 |
| Liverpool | 43 | 38 | 1.13 | 25 | 20 | 1.25 |
| Ushant | n/a | n/a | n/a | 17 | 16 | 1.06 |
| Quiberon | 44 | 33 | 1.33 | n/a | n/a | n/a |
| Scillies | 46 | 39 | 1.18 | n/a | n/a | n/a |
| TOTAL | 486 | 411 | 1.18 | 232 | 20.3 | 1.14 |

Table S10. Predicted proportions (%) of pathogen prevalence for three bee species when *Varroa* is present and absent (conversion of GLMM (table S8) estimates on the logit scale to proportions using the formula exp(x)/(1+exp(x)), where x equals the parameter estimate

| Response | Species | Prevalence when *Varroa* absent | Prevalence when *Varroa* present |
| --- | --- | --- | --- |
| DWV-B | *A. mellifera* | 9.53 | 68.78 |
|  | *B. pascuorum* | 0.62 | 11.61 |
|  | *B. terrestris* | 0.36 | 6.98 |

Table S11. Best model explaining *Nosema* (*N. bombi* and *N.ceranae*) prevalence (GLMMs with binomial error structure and logit link function). The predictor state of the intercept is *A. mellifera,* on a *Varroa*-free site. Population of origin was included as a random effect

| Response | Predictors | Estimate | SE | z-value | P-value |
| --- | --- | --- | --- | --- | --- |
| a) *Nosema* prevalence | Intercept | -42.13 | 10.97 | -3.84 | <0.001 |
|  | *B. pascuorum* | -1.12 | 0.79 | -1.41 | 0.16 |
|  | *B. terrestris* | 1.58 | 0.70 | 2.27 | 0.02 |
|  | Island site  *Varroa* presence | -1.37 | 0.80 | -1.71 | 0.09 |
|  |  | -1.16 | 0.82 | -1.41 | 0.16 |
|  | Sunshine hours (log) | 18.36 | 4.81 | 3.82 | <0.001 |

Table S12. Population genetics for DWV-B by individual fragment: Kst and Snn are calculated to determine if populations are structured by either host or geographic location. Bold text indicates significance.

| Fragment | Trait | *Kst* | - - - 1. *p_Kst_* | *Snn* | - - - 1. *p_Snn_* |
| --- | --- | --- | --- | --- | --- |
| *lp* | host | 0.002 | >0.05 | 0.710 | >0.05 |
| ***lp*** | **location** | **0.206** | **<0.001** | **0.408** | **<0.001** |
| *vp3* | host | 0.002 | >0.05 | 0.522 | >0.05 |
| ***vp3*** | **location** | **0.072** | **<0.001** | **0.19** | **<0.001** |
| *helicase* | host | -0.005 | >0.05 | 0.412 | >0.05 |
| ***helicase*** | **location** | **0.062** | **<0.001** | **0.245** | **<0.001** |
| *rdrp* | host | 0.003 | >0.05 | 0.696 | >0.05 |
| ***rdrp*** | **location** | **0.079** | **<0.001** | **0.286** | **<0.001** |
| - - - 1. concatenated | host | - - - 1. 0.005 | - - - 1. >0.05 | - - - 1. 0.896 | - - - 1. >0.05 |
| - - - 1. **concatenated** | **location** | **0.245** | **<0.001** | **0.657** | **<0.001** |

Table S13. Mean root heights and exponential growth with 95% HPD (Highest posterior density) in brackets, estimated from Beast 1.8 models for concatenated and individual fragments.

| Fragment | Partitions | Mean exp. growth rate | Doubling rate [years] | - - - 1. Mean root height [years] |
| --- | --- | --- | --- | --- |
| - - - 1. Concat 4 | - - - 1. host and site | - - - 1. 0.95 (0.29 - 1.76) | - - - 1. 0.73 (0.39 - 2.39) | - - - 1. 5.7 (2.0 - 10.3) |
| - - - 1. *lp* | - - - 1. no traits | - - - 1. 0.79 (0.16 - 1.59) | - - - 1. 0.88 (0.44 – 4.33) | - - - 1. 6.7 (2.1 - 12.8) |
| - - - 1. *Vp3* | - - - 1. no traits | 0.89 (0.22 - 1.81) | - - - 1. 0.78 (0.38 – 3.15) | - - - 1. 6.8 (2.0 - 12.9) |
| - - - 1. *helicase* | - - - 1. no traits | - - - 1. 1.11 (0.13 - 2.39) | - - - 1. 0.62 (0.29 – 5.33) | - - - 1. 4.8 (1.5 - 9.6) |
| - - - 1. *rdrp* | - - - 1. no traits | - - - 1. 1.50 (0.32 - 2.95) | - - - 1. 0.46 (0.23 - 2.17) | - - - 1. 4.0 (1.3 - 7.6) |

*Supplementary figures*


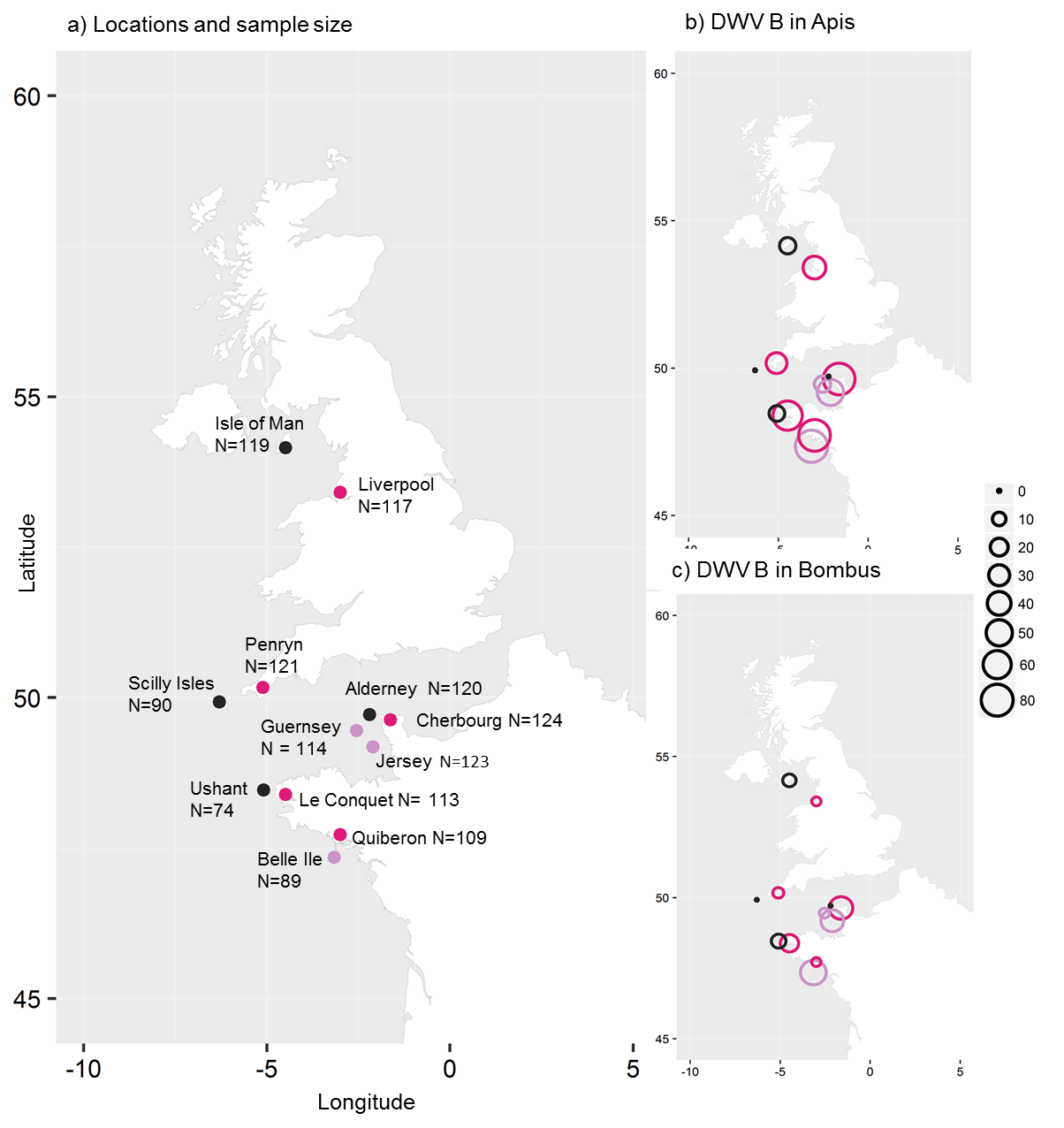


Figure S1. Sampling locations and sample sizes


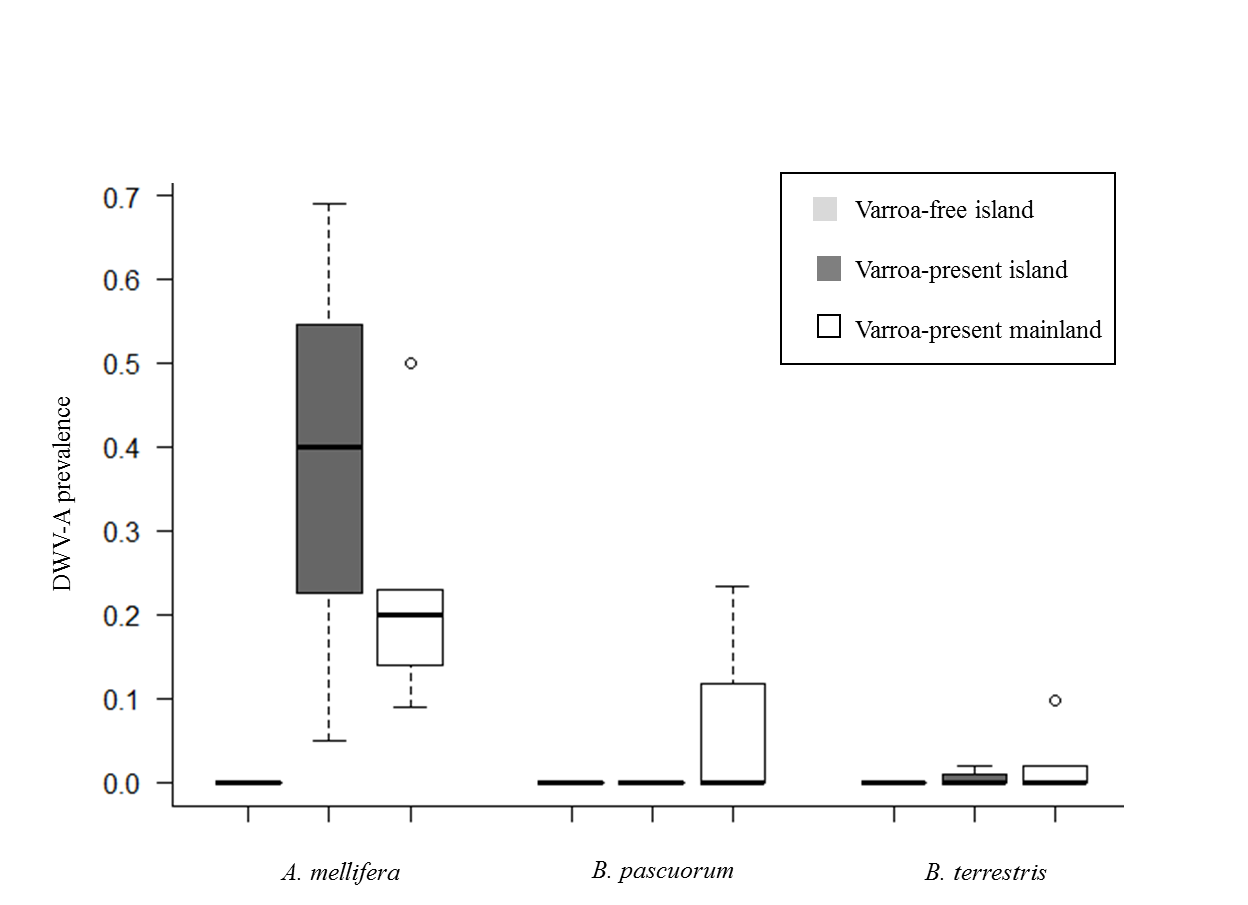


Figure S2. Prevalence (with 95% confidence intervals) of DWV-A by host species and *Varroa*-free (V-, light grey), *Varroa*-present islands (dark grey) and *Varroa*-present mainland sites (V+M, white).


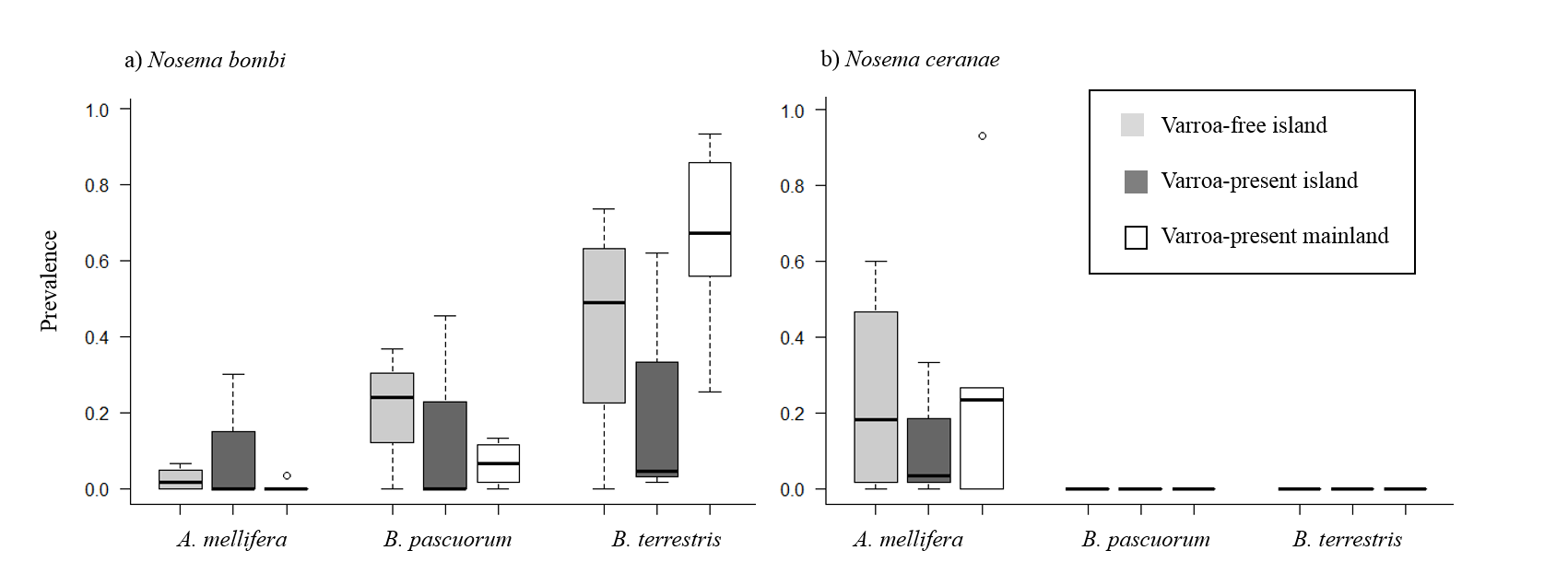


Figure S3. Prevalence (with 95% confidence intervals) of *Nosema bombi* (a) and *N. ceranae* (b) by host species and *Varroa*-free (V-, light grey), *Varroa*-present island (dark grey) and *Varroa*-present mainland sites (V+M, white).

Figure S4. Individual fragment phylogenetic trees (a) *Lp* b) *Vp3* c) *helicase* d) *RdRp*) produced using MrBayes analysis with all major nodes showing high posterior support >0.8. Viral sequences from bumblebee samples are indicated with *, all others originate from honeybees. An orange star identifies samples that came from *Varroa-*free locations, all others originate from *Varroa-*present island and mainland locations.


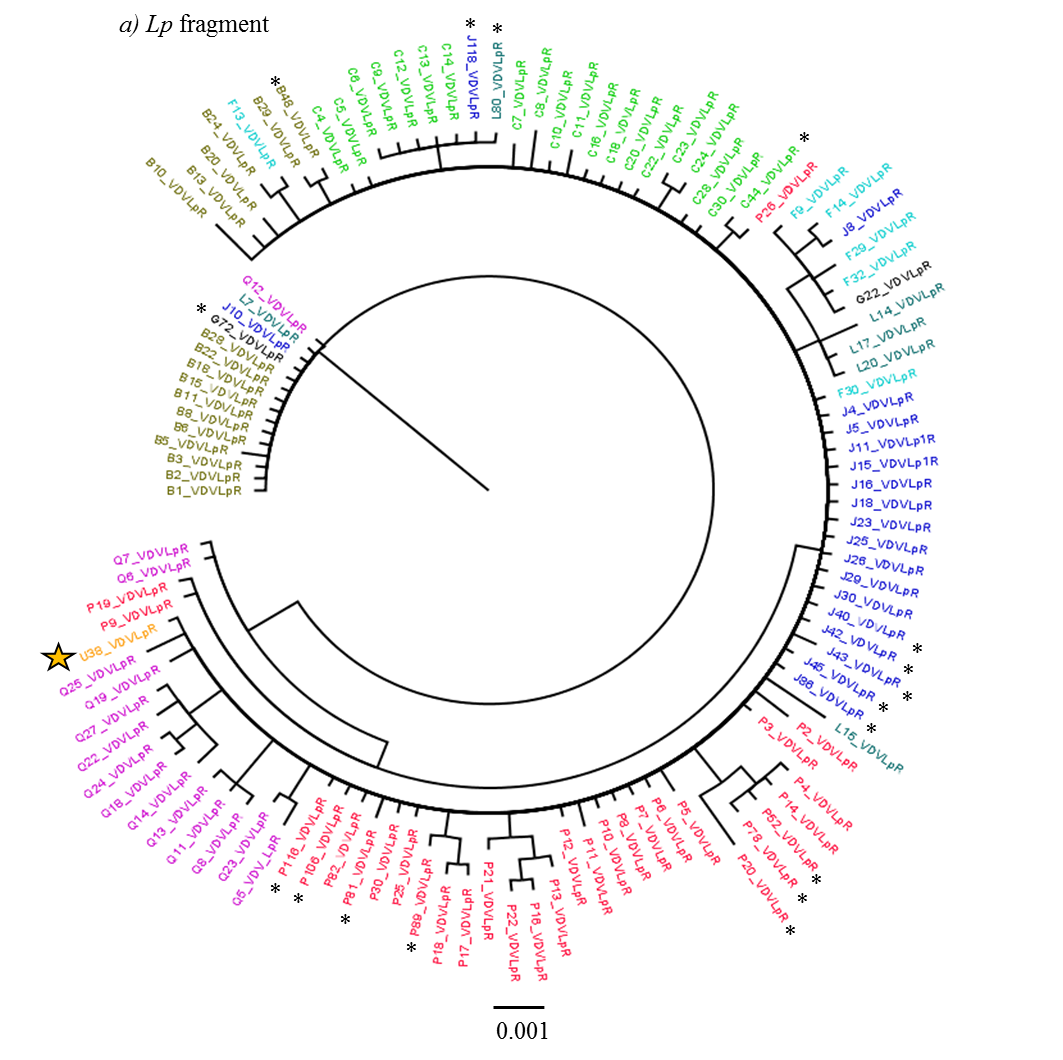


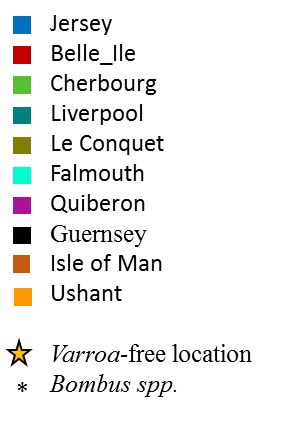


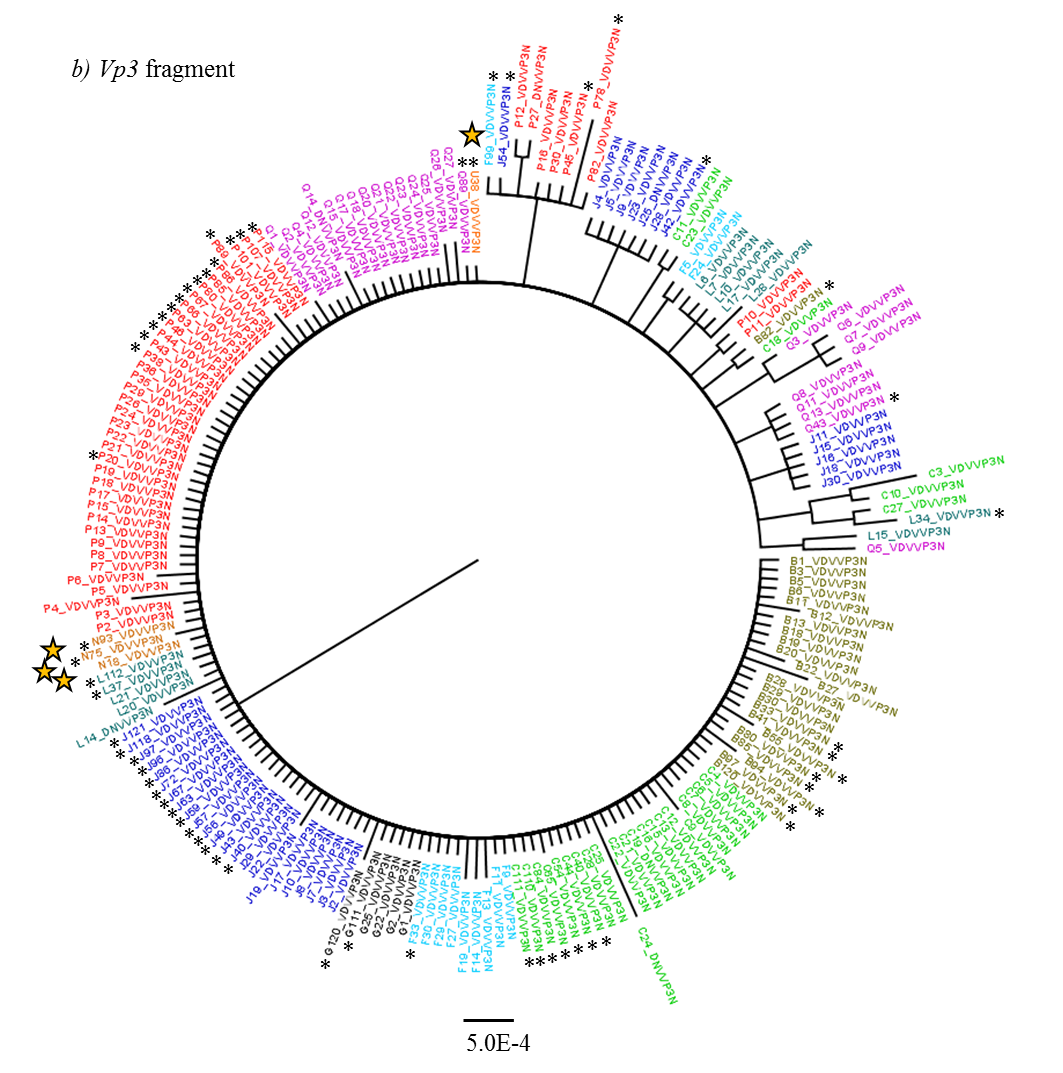


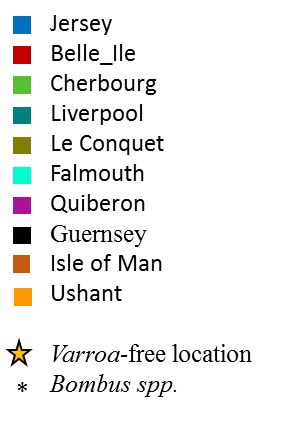


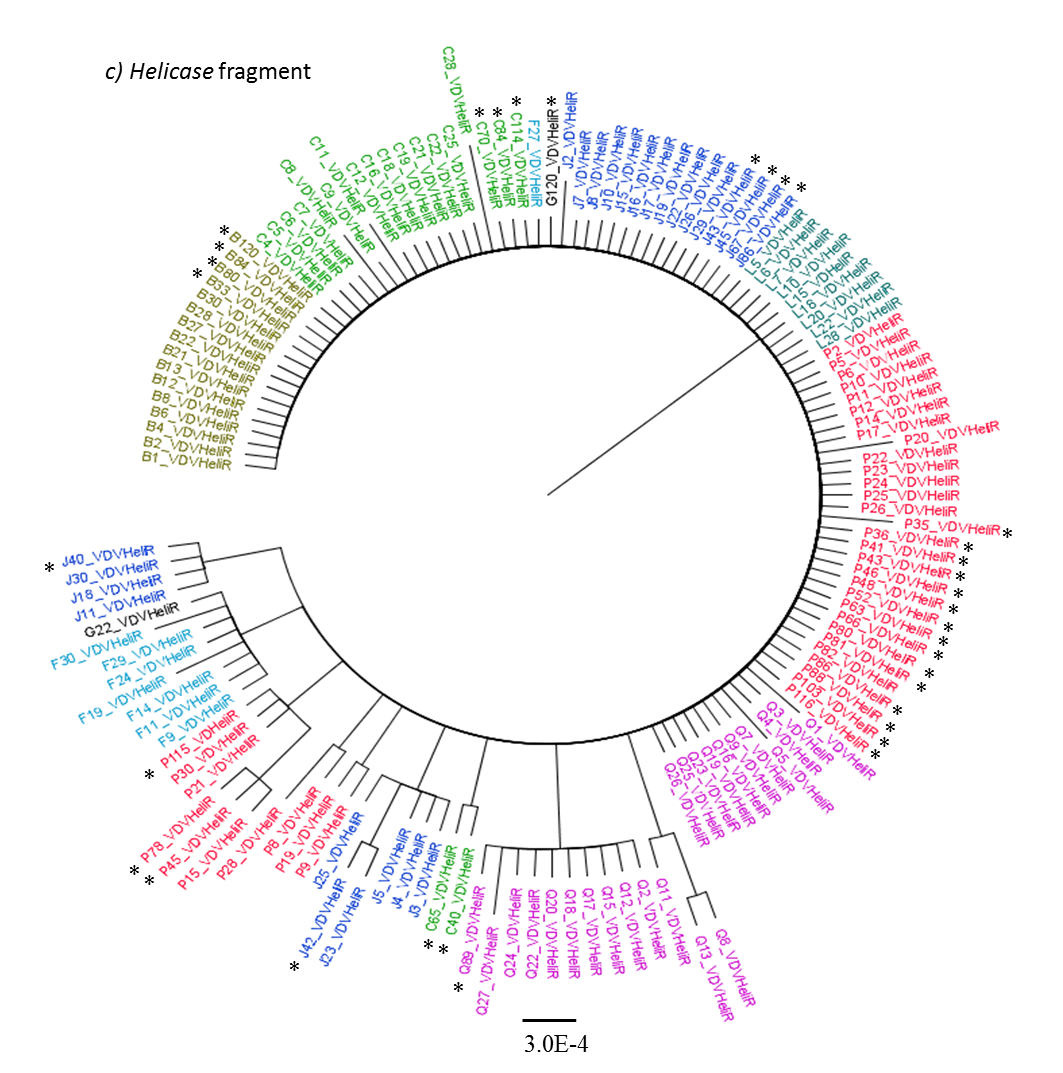


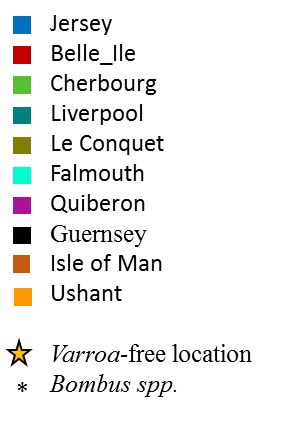


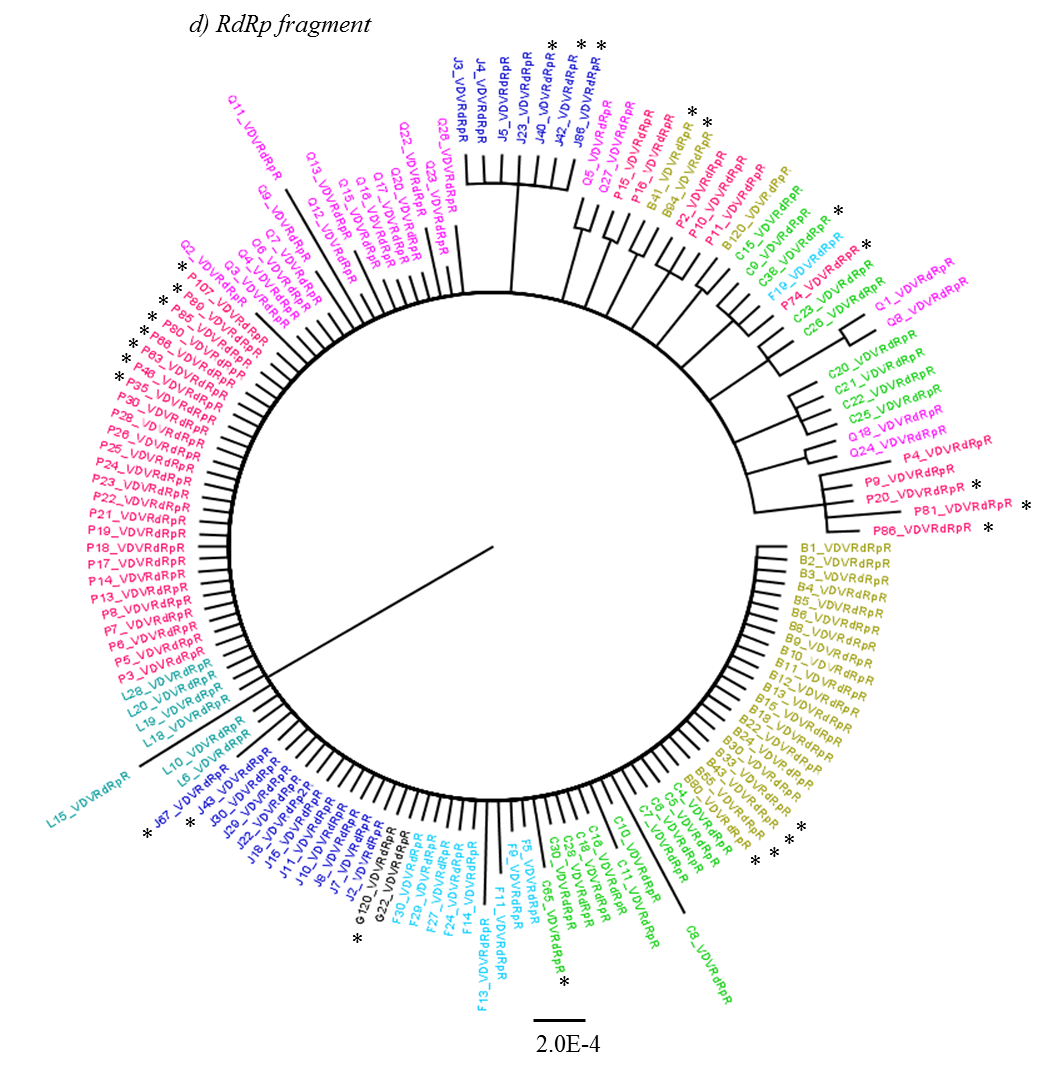


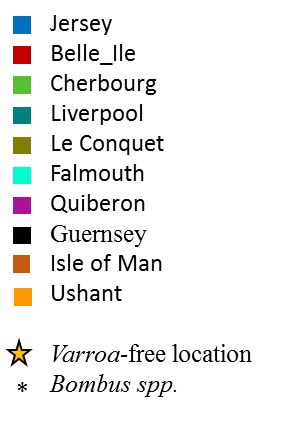

Figure S5. Maximum clade credibility (MCC) tree for Deformed wing virus (type B) (genomes comprised of four concatenated fragments of DWV-B; regions of the *lp*-, *vp3*-, *helicase*- and *rdrp*-gene fragments, total length = 1108 bp) showing host and location structure. Branches are coloured according to host species (*A. mellifera* in red, and *B. terrestris* in blue), and the branch tips are coloured by location (see key). Posterior support >0.5 is indicated for nodes up to the 3rd order. The scale is time, in years.

Figure S6. Five DWV reads from SMRT sequencing of *A. mellifera* pool collected from Liverpool, identified as potential recombinants within the *rdrp*-gene: DWV-A (red) and DWV-B (blue). The reads in the panels a-c show convincing evidence of recombination.

**Supplementary methods**

S1. Microsatellite methods and analysis

DNA was extracted from a tarsal sample removed from the individuals, using Chelex® (following methods in Walsh *et al.* 1991). The tarsal samples were crushed in liquid nitrogen using sterile pestles before adding 200µl of 5% chelex solution and 2µl of Proteinase K, vortexed and heated to 56^o^C for an hour and then 96^o^C for 15minutes. We centrifuged the samples at maximum RPM for 1 minute, and 150µl of the supernatant was stored at -20^o^C. The extracted DNA was amplified at 9 polymorphic microsatellite loci (Estoup *et al.* 1995; Estoup *et al.* 1996). The same nine markers were used for both species (*B100, B118, B132, B10, B11, B96, B119, B124, B126*), when *B100* was replaced with *B121* and *B119* replaced with *B131* for *B. pascuorum*. Locus *B121* and *B118* were dropped from *B. pascuorum* analysis due to poor amplification across all field sites. We ran three multiplex PCRs as detailed in the table below following methods from Dreier *et al.* (2014) with 1µL of template DNA. We diluted the PCR products 1:10 and visualised them on an ABI 3730 capillary DNA sequencer using GeneScan Liz 500 size standard. We scored fragments using Geneious version 9.1.5.

| Primer Mix | Optimum Annealing Temperature (T_m_) | PCR cycles |
| --- | --- | --- |
| *B100*(VIC)/*B121*(VIC) –*B118*(NED)-*B132*(FAM), | 58^o^C | 15 m at 95C  [30 s at 94C  90 s at T_m_  60 s at 72C]  X25tttttttttt  45 m 60C |
| *B10*(VIC)-*B11*(NED)-*B96*(FAM) | 52^o^C |  |
| *B119*(FAM)/*B131*(FAM)-*B124*(NED)-*B126*(PET) | 56^o^C |  |

MICRO-CHECKER (Van Oosterhout *et al.* 2004) was used to identify microsatellite genotyping errors by analysing each locus for homozygote excess, which serves as an indicator for the presence of null alleles. COLONY 2.0.6.4 (Wang 2004; Jones & Wang 2010) was used to identify sister individuals within each site. Samples were identified to be from the same colony on the basis of a posterior probability of > 0.8, following methods from the literature (e.g. Carvell *et al.* 2012; Carvell *et al.* 2017). We assumed that all workers within a colony were offspring of one singly-mated queen (Estoup *et al.* 1995; Schmid-Hempel & Schmid-Hempel 2000) and genotyping error rates of 0.05% for *B. terrestris* and 0.05% for *B. pascuorum* of all samples.”

S2. Plasmids for qPCR

DWV-A and DWV-B plasmids were generated using Promega pGEM®-T Easy Vector, according to the manufacturer’s instructions, to clone a 360bp fragment and 89bp fragment of the *rdrp*-gene in DWV-A and -B, respectively, from purified PCR products (primer details in table S8). Successful transformants were selected via blue/white screening, and plasmids were extracted using the GeneJET Plasmid Miniprep Kit (ThermoFisher). M13 primers (designed to sequence inserts inside pGEM®-T Easy Vector: forward 5´- GTTTTCCCAGTCACGAC 3´, reverse 5´-CAGGAAACAGCTATGAC -3´) were used to verify that the correct product had been cloned. We linearised the plasmids using the restriction enzyme *Apa1* (New England Biolabs), according to the manufacturer’s instructions, and diluted them 1:1000 with RNase-free water. Mean efficiency across plates for DWV-A was 90.6% (four plates with range 89.2 - 92.1%) and DWV-B was 92.2% (seven plates ranging from 90.8 - 92.2%) with R2 > 0.98 across assays.

S3. Negative-strand detection by RT-qPCR

DWV is a positive strand virus whose negative strand is only present during virus replication, thus detection of the negative strand is a strong indicator of a true infection. To demonstrate that DWV-A and DWV-B are replicating across our samples, specifically in *Bombus* samples, we tested all positive *Bombus* samples (DWV-A, n = 18, DWV-B, n = 49) and a randomly chosen sub-set of *Apis* samples (DWV-A, n = 10, DWV-B n =10) for replication. We conducted reverse transcription with tagged forward primer DWV_F2_TAG agcctgcgcaccgtggTGTCTTCATTAAAGCCACCTGGAA and VDV_F2_TAG agcctgcgcaccgtggTATCTTCATTAAAACCGCCAGGCT (primers adapted from (Fürst *et al.* 2014; McMahon *et al.* 2015)) to target the negative strand exclusively. The resulting tagged-cDNA was treated with Exonuclease 1 (0.5ul per sample at 37ºC for 40 minutes, followed by 80 ºC for 15 minutes) and used in qPCR with the tag sequence and DWV-R2a or VDV-R2a respectively (table S8). This negative strand assay was run alongside the original positive strand assay and plasmid standard curves (table S8). We ran controls at the reverse transcription and qPCR stages to minimise false detection of the negative strand: cDNA was made with no primer present to check against self-priming; we conducted a reverse primer only qPCR, and included no template controls and known negative sample controls. Amplification and melt curves were analysed to determine the presence or absence of negative strand.

S4. Recombination

1. To confirm that there was no recombination within fragments at a p value of 0.05, we used the GENECONV (Padidam *et al.* 1999), MaxChi (Maynard Smith 1992), BootScan (Martin *et al.* 2005) and SiScan (Gibbs *et al.* 2000) algorithms in the Rdp4 package (v4.56) (Martin *et al.* 2015). Only the *lp*-gene fragment contained recombinants (N = 13), and these we removed for subsequent phylogenetic analysis. We found two points of recombination within the *lp* region: one at ~1550 and one ~1660, with both DWV-A and DWV-B seen at the 5’ and 3’ end of the sequenced fragment.
2. Pacbio reads mapping to the genomes of DWV-A, B and C were extracted for further analysis. To identify recombinants, reads mapping to known DWV variants from each pool were fragmented into 100bp windows with a 50bp overlap (windows of 200-2000nt were also tested, but resulted in reduced sensitivity in the detection of synthetic *in silico* recombinants). Windows were then mapped back to known DWV genomes using bbmap (<https://github.com/BioInfoTools/BBMap>) to identify the best mapping for each window. Putative recombinants were identified when three consecutive windows (150bp) aligned to a different variant compared to the rest of the windows from the parent read.

S5. Phylogenetic reconstruction: model selection, evolutionary rates and migration routes

For individual and concatenated DWV-B fragment alignments (table S4), we used Jmodeltest (v.2.1) (Guindon & Gascuel 2003a; Darriba *et al.* 2012b) to compare evolutionary substitution models based on the Bayesian Information Criterion (Alizon & Fraser 2013). In Beast 1.8, we partitioned substitution rates between the 1^st^ & 2^nd^ and 3^rd^ codon positions. For the concatenated alignments we fit discrete trait models with asymmetric substitution models for host species and geographic location (note, there was insufficient genetic information in the individual fragment alignments to include traits). The evolutionary rate prior was set at 1.35 x 10^-3^ changes per site per year (95% HPD 5.41 x 10^-4^ - 2.63 x 10^-3^), with a lognormal distribution and standard deviation of 0.4(Mordecai *et al.* 2015) to estimate time since divergence from a common ancestor. This rate was chosen as there was little temporal information in the currently available DWV-B sequences; only the *lp*-fragment showed a weak temporal signal (correlation coefficient with best fitting root 0.27, R_2_ = 0.07) based on a MrBayes-generated phylogenetic tree analysed in TempEst (Rambaut *et al.* 2016). There was no signal for the *vp3-* or *rdrp-*fragments (correlation coefficients -0.12 and -0.19, R_2_ = 0.012 and 0.0035 respectively). For the *lp*-fragment, we estimated the evolutionary clock using a lognormal relaxed clock and an exponential growth prior, based on our standard model selection procedure. We used the SRD06 model of sequence evolution (Shapiro *et al.* 2006). Based on this procedure, the clock rate for DWV-B was estimated as 1.99 x10^-3^ per bp per year (95% HPD 4.58 x10^-4^ to 4.07 x10^3^); this overlaps with the 95% HPD of the mean DWV-A clock mean so we therefore chose to use the better supported prior previously estimated from DWV-A (Mordecai *et al.* 2015). We compared nine models per fragment with different demography (constant population size, exponential growth and GMRF Bayesian Skyride) and molecular clock rates (strict, relaxed lognormal and exponential clocks), and used the path sampling maximum likelihood estimator, implemented in Beast 1.8, to determine the best model (table S10). All models were run long enough to obtain effective sample sizes >200 for all parameters, with a 10% burn-in, sampling the chain at equal distances to obtain a total of 10,000 trees per analysis. We checked models for convergence in Tracer (v1.6), determined if a strict clock could be excluded or not by examining the relaxed lognormal clock’s coefficient of variation statistic, and for models with an exponential growth prior, we determined if there was significant exponential growth (Drummond & Bouckaert 2015).

# References

Alizon, S. & Fraser, C. (2013). Within-host and between-host evolutionary rates across the HIV-1 genome. *Retrovirology*, 10, 49 DOI: 10.1186/1742-4690-10-49.

Berenyi, O., Bakonyi, T., Derakhshifar, I., Koglberger, H., Topolska, G., Ritter, W. *et al.* (2007). Phylogenetic analysis of Deformed Wing Virus genotypes from diverse geographic origins indicates recent global distribution of the virus. *Applied and Environmental Microbiology*, 73, 3605-3611 DOI: 10.1128/aem.00696-07.

Carvell, C., Bourke, A.F., Dreier, S., Freeman, S.N., Hulmes, S., Jordan, W.C. *et al.* (2017). Bumblebee family lineage survival is enhanced in high-quality landscapes. *Nature*, 543, 547-549.

Carvell, C., Jordan, W.C., Bourke, A.F., Pickles, R., Redhead, J.W. & Heard, M.S. (2012). Molecular and spatial analyses reveal links between colony‐specific foraging distance and landscape‐level resource availability in two bumblebee species. *Oikos*, 121, 734-742.

Darriba, D., Taboada, G.L., Doallo, R. & Posada, D. (2012a). jModelTest 2: more models, new heuristics and parallel computing. *Nature Methods*, 9, 772-772 DOI: 10.1038/nmeth.2109.

Darriba, D., Taboada, G.L., Doallo, R. & Posada, D. (2012b). jModelTest 2: more models, new heuristics and parallel computing. *Nature Methods*, 9, 772 DOI: 10.1038/nmeth.2109.

de Miranda, J. (2008). Diagnostic techniques for virus detection in honey bees. In: *Virology and the honey bee* (eds. Aubert, M, Ball, BV, Fries, I, Moritz, RFA, Milani, N & Bernardelli, I). European Communities Luxembourg, pp. 121-232.

Dreier, S., Redhead, J.W., Warren, I.A., Bourke, A.F., Heard, M.S., Jordan, W.C. *et al.* (2014). Fine‐scale spatial genetic structure of common and declining bumble bees across an agricultural landscape. *Molecular Ecology*, 23, 3384-3395.

Drummond, A.J. & Bouckaert, R.R. (2015). *Bayesian Evolutionary Analysis with BEAST*. Cambridge University Press.

Estoup, A., Solignac, M., Cornuet, J.M., FGoudet, J. & Scholl, A. (1996). Genetic differentiation of continental and island populations of Bombus terrestris (Hymenoptera: Apidae) in Europe. *Molecular Ecology*, 5, 19-31.

Estoup, A., Tailliez, C., Cornuet, J.M. & Solignac, M. (1995). Size homoplasy and mutational processes of interrupted microsatellites in two bee species, Apis mellifera and Bombus terrestris (Apidae). *Molecular Biology and Evolution*, 12, 1074-1084.

Fries, I., Chauzat, M.-P., Chen, Y.-P., Doublet, V., Genersch, E., Gisder, S. *et al.* (2013). Standard methods for Nosema research. *Journal of Apicultural Research*, 52 DOI: 10.3896/ibra.1.52.1.14.

Fürst, M.A., McMahon, D.P., Osborne, J.L., Paxton, R.J. & Brown, M.J.F. (2014). Disease associations between honeybees and bumblebees as a threat to wild pollinators. *Nature*, 506, 364-366 DOI: <https://doi.org/10.1038/nature12977>.

Genersch, E. (2005). Development of a rapid and sensitive RT-PCR method for the detection of deformed wing virus, a pathogen of the honeybee (*Apis mellifera*). *Veterinary Journal*, 169, 121-123 DOI: 10.1016/j.tvjil.2004.01.004.

Gibbs, M.J., Armstrong, J.S. & Gibbs, A.J. (2000). Sister-Scanning: a Monte Carlo procedure for assessing signals in recombinant sequences. *Bioinformatics*, 16, 573-582.

Guindon, S. & Gascuel, O. (2003a). A simple, fast and accurate method to estimate large phylogenies by maximum-likelihood. *Systematic Biology*, 52, 696-704.

Guindon, S. & Gascuel, O. (2003b). A simple, fast, and accurate algorithm to estimate large phylogenies by maximum likelihood. *Syst. Biol.*, 52, 696-704 DOI: 10.1080/10635150390235520.

Jones, O.R. & Wang, J. (2010). COLONY: a program for parentage and sibship inference from multilocus genotype data. *Molecular Ecology Resources*, 10, 551-555.

Martin, D.P., Murrell, B., Golden, M., Khoosal, A. & Muhire, B. (2015). RDP4: Detection and analysis of recombination patterns in virus genomes. *Virus Evolution*, 1, vev003 DOI: 10.1093/ve/vev003.

Martin, D.P., Posada, D., Crandall, K.A. & Williamson, C. (2005). A modified bootscan algorithm for automated identification of recombinant sequences and recombination breakpoints. *AIDS Research and Human Retroviruses*, 21, 98-102.

Maynard Smith, J. (1992). Analyzing the mosaic structure of genes. *Journal of Molecular Evolution*, 34, 126-129.

McMahon, D.P., Fürst, M.A., Caspar, J., Theodorou, P., Brown, M.J.F. & Paxton, R.J. (2015). A sting in the spit: widespread cross-infection of multiple RNA viruses across wild and managed bees. *Journal of Animal Ecology*, 84, 615-624.

Mordecai, G.J., Wilfert, L., Martin, S.J., Jones, I.M. & Schroeder, D.C. (2015). Diversity in a honey bee pathogen: first report of a third master variant of the Deformed Wing Virus quasispecies. *ISME J* DOI: 10.1038/ismej.2015.178.

Padidam, M., Sawyer, S. & Fauquet, C.M. (1999). Possible emergence of new Geminiviruses by frequent recombination. *Virology*, 265, 218-225.

Pascall, D.J., Tinsley, M.C., Obbard, D., J, & Wilfert, L. (2018). Host evolutionary history predicts virus prevalence across bumblebee species *BioRxiv* DOI: <https://doi.org/10.1101/498717>.

Rambaut, A., Lam, T.T., Carvalho, M.L. & Pybus, O.G. (2016). Exploring the temporal structure of heterochronous sequences using TempEst (formerly Patho-O-Gen). *Virus Evolution*, 2 DOI: 10.1093/ve/vew007.

Schmid-Hempel, P. & Schmid-Hempel, R. (2000). Female mating frequencies in *bombus* spp. from Central Europe. *Insectes Sociaux*, 47, 36-41.

Shapiro, B., Rambaut, A. & Drummond, A.J. (2006). Choosing appropriate substitition models for the phylogenetic analysis of protein coding sequences. *Molecular Biology and Evolution*, 23, 7-9.

Tay, W.T., O'Mahoney, E.M. & Paxton, R.J. (2005). Complete rRNA gene sequences reveal that the microsporidium Nosema bombi infects diverse bumble bee (Bombus spp.) hosts and contains multiple polymorphic sites. *Journal of Eukaryotic Microbiology*, 52, 505-513.

Van Oosterhout, C., Hutchinson, W.F., Wills, D.P. & Shipley, P. (2004). MICRO‐CHECKER: software for identifying and correcting genotyping errors in microsatellite data. *Molecular Ecology Notes*, 4, 535-538.

Walsh, P.S., Metzger, D.A. & Higuchi, R. (1991). Chelex 100 as a medium for simple extraction of DNA for PCR-based typing from forensic material. *Biotechniques*, 10, 506-513.

Wang, J. (2004). Sibship reconstruction from genetic data with typing errors. *Genetics*, 166, 1963-1979.

Wilfert, L., Long, G., Leggett, H.C., Schmid-Hempel, P., Butlin, R., Martin, S.J.M. *et al.* (2016). Deformed wing virus is a recent global epidemic in honeybees driven by *Varroa* mites. *Science*, 351, 594-597 DOI: 10.1126/science.aac9976.

Yue, C. & Genersch, E. (2005). RT-PCR analysis of *Deformed wing virus* in honeybees (*Apis mellifera*) and mites (*Varroa destructor*). *Journal of General Virology*, 86, 3419-3424.
